# Peritoneal dialysis prescription in children: bedside principles for optimal practice

**DOI:** 10.1007/s00467-008-0979-7

**Published:** 2009-09-01

**Authors:** Michel Fischbach, Bradley A. Warady

**Affiliations:** 1 Pediatry 1, University Hospital, Avenue Molière, 67098 Strasbourg Cedex, France; 2 Section of Pediatric Nephrology, Children’s Mercy Hospital, 2041 Gillham Road, Kansas City, MO 64108–4698 USA

**Keywords:** Peritoneal dialysis, Children, Fill volume, Dwell time, Peritoneal membrane, Icodextrin

## Abstract

There is no unique optimal peritoneal dialysis prescription for all children, although the goals of ultrafiltration and blood purification are universal. In turn, a better understanding of the physiology of the peritoneal membrane, as a dynamic dialysis membrane with an exchange surface area recruitment capacity and unique permeability characteristics, results in the transition from an empirical prescription process based on clinical experience alone to the potential for a personalized prescription with individually adapted fill volumes and dwell times. In all cases, the prescribed exchange fill volume should be scaled for body surface area (ml/m^2^), and volume enhancement should be conducted based on clinical tolerance and intraperitoneal pressure measurements (IPP; cmH_2_O). The exchange dwell times should be determined individually and adapted to the needs of the patient, with particular attention to phosphate clearance and ultrafiltration capacity. The evolution of residual kidney function and the availability of new, more physiologic, peritoneal dialysis fluids (PDFs) also influence the prescription process. An understanding of all of these principles is integral to the provision of clinically optimal PD.

The peritoneal membrane is a dialysis membrane for the peritoneal dialysis (PD) patient. Ideally, the permeability of the peritoneum and the surface area membrane recruitment capacity [1] should be determined as part of the prescription process. Knowledge about the contact surface area recruitment capacity [2, 3], the so called “wetted” membrane, and vascular surface area changes [4, 5], is important because of the desire to prevent hyperperfusion of the peritoneal membrane, as it may contribute to the development of membrane failure [6–8].

Nevertheless, in practice, greater significance is given to the following clinical parameters [9]:

- Choice of peritoneal dialysis fluid (PDF) [10], with particular reference to dextrose concentration (and the associated ability to meet ultrafiltration needs) and its biocompatibility for peritoneal membrane preservation
- Tolerance of the prescribed fill volume, determined by patient report, at times with the assistance of intraperitoneal pressure (IPP) measurement [11]
- Dwell time of dialysis exchanges adapted to the individual patient’s needs [12]

All these factors play key roles in achieving adequate PD.

Finally, it must be emphasized that the PD prescription should be individualized and adapted to achieve at least two main goals: (1) adequate ultrafiltration to avoid hypervolemia because of its contribution to cardiovascular morbidity and mortality, and (2) blood purification of solute, not limited solely to urea [9, 13].

## Peritoneal dialysis principles: physiology of the peritoneal membrane

The peritoneal membrane is a dynamic dialysis membrane [1, 14, 15]. The actual surface area that is in contact with the dialysate is only 30–60% of the available anatomic area in humans as measured by computed tomography [2, 3]. The size of the contact area is dependent upon a variety of factors—including posture, with positive recruitment occurring in the supine position—and fill volume, with a progressive increase in recruitment taking place until the fill volume is close to 1,400 ml/m^2^ body surface area (BSA) in children 2 years of age and older [4]. Peritoneal vascular mesenteric perfusion and density of the functional pores of the perfused capillaries determine the peritoneal vascular exchange surface area [1, 15, 16]. This area is dynamically affected by different factors, such as PDF composition, fill volume size, and possibly inflammatory agents [4–8].

Physiology of the dialysis process through the peritoneal barrier is complex [14, 15]. One of the most influential factors is capillary permeability, described by the three-pore model that addresses both the diffusive process and convective mass transport [16]. The ultrasmall pores, aquaporins, are involved in sodium-free water exchange, the small pores (the most numerous pores) allow diffusion to take place, and the large pores facilitate convective mass transport. The concentration gradient of a solute between the plasma and the dialysate, which is in contact with the peritoneal surface area, along with solute-specific peritoneal membrane permeability coefficient, are the determinants of the solute diffusion capacity. Low molecular weight compounds such as urea are preferentially cleared by the diffusive exchange purification process. In contrast, a pressure gradient governs ultrafiltration and the convective mass transport of high molecular weight compounds such as the “middle molecular weight” uremic toxins [9, 13]. This pressure gradient is the arithmetic sum of hydrostatic and osmotic pressures. The hydrostatic pressure gradient results from intravascular pressure and IPP. Osmotic pressure could be the result of either crystalloid (i.e. generated by diffusible solutes such as glucose in the dialysate) or colloid (i.e. generated by nondiffusible solutes such as albumin in the plasma or icodextrin in the dialysate). Currently, the osmotic agent used most frequently in PDFs is dextrose, and the associated osmotic crystalloid generated gradient is only transient: it decreases secondary to the time-dependent diffusion of glucose from the dialysate to the plasma. Conversely, icodextrin is a polymer of glucose that is secondarily hydrolyzed into maltose and has colloid osmotic properties, which allows for the icodextrin-generated osmotic gradient to be more sustained [10, 17–20].

## Choice of peritoneal dialysis fluid

The peritoneal membrane is in contact with the PDF; therefore, biocompatibility is of great importance for maintaining PD as a viable long-term dialysis modality [1, 19]. This is an especially pertinent issue for the pediatric patient. In automated peritoneal dialysis (APD), the most commonly prescribed dialysis modality in children, contact between the PDF and the peritoneal membrane is repeated frequently over the course of a daily dialysis session, a fact that may increase the significance of the solution’s biocompatibility. A variety of PDF-related factors has been implicated in the bioincompatibility process and may result in deleterious effects on the peritoneal structure (diabetiform vascular changes) and function (ultrafiltration failure, purification loss). These factors include glucose degradation products (GDPs), dialysate pH, buffer type (lactate in a supraphysiologic concentration), and osmolality (glucose concentration). The glucose used as an osmotic agent is in a nonphysiological concentration, and thereby results in direct toxicity. However, it is the GDPs generated by the heat sterilization process that are the primary determinants of peritoneal toxicity. The GDPs result in an increased membrane thickness and, as noted above, a “diabetiform” vasculopathy, initially with neoangiogenesis (hyperpermeability), and thereafter with fibrosis (hypopermeability) and ultrafiltration failure. GDPs can also cross-link with proteins and produce advanced glycosylated end products (AGEs), which have been implicated in the loss of residual kidney function [19]. High osmolality, acidity, and/or a high lactate content have been associated with deleterious effects on leucocyte and mesothelial cell function.

New PDFs, the development of which has taken prior concerns into consideration, are now available [10, 19–21] (Tables 1 and 2). It should be emphasized, however, that no single PDF can necessarily achieve all of the dialysis prescription goals, with optimal ultrafiltration and optimal blood purification being the desired clinical targets. These new, more physiologic, solutions differ from the conventional ones in their production process, as reflected by the development of two-compartment dialysis bags, and in their composition with respect to the buffer agent, pH, osmotic agent, and their GDP concentration. All new PDFs offer enhanced biocompatibility by reducing GDP concentration. Despite the availability of these solutions in some countries, there is no consensus regarding the best PDF to prescribe and whether bicarbonate is in fact the optimal buffer for all new PDFs [6]. In children, the application of pH-neutral solutions with a low concentration of GDPs and bicarbonate or a bicarbonate/lactate mixture as buffer, has been associated with better membrane preservation and improved mesothelial cell function, as reflected by an increased dialysate concentration of CA125 (see below) [7, 8, 22]. The pH-neutral bicarbonate-based solutions appear to have superior buffering capacity compared with a lactate-based dialysis fluid in children undergoing APD [8]. The nearly neutral pH PDFs have been shown to induce less pain at peritoneal filling and are associated with a smaller increase in IPP for the same amount of fill volume [5, 8, 11]. The impact of the improved biocompatibility on leukocyte function and peritoneal defense requires additional study [19].

**Table 1:** Characteristics of currently available multibag peritoneal dialysis (PD) solutions [19]

	Manufacturer	Potential drawbacks	Potential benefits
Lactate-buffered: Balance®, Gambrosol Tri®	Fresenius Gambro	More physiological pH, but not neutral. Local and systemic glucose exposure	Lower GDP levels
			More physiological pH
			Improved peritoneal membrane biocompatibility
			Preserved membrane defense
Lactate/bicarbonate-buffered: Physioneal®	Baxter	Local and systemic glucose exposure. Does not eliminate peritoneal lactate exposure	Lower GDP levels
			More physiological pH
			Improved peritoneal membrane biocompatibility
			Preserved membrane defense
			Reduced infusion pain
Bicarbonate-buffered: BicaVera®	Fresenius	Local and systemic glucose exposure	Lower GDP levels
			More physiological pH
			More peritoneal membrane biocompatibility
			Preserved membrane defense
			Improved correction of acidosis

*GDP* glucose degradation product

**Table 2:** Characteristics of currently available single-bag peritoneal dialysis (PD) solutions [19]

	Manufacturer	Potential drawbacks	Potential benefits
Lactate-buffered glucose containing Dianeal®	Baxter	Low pH	Ease of manufacture; low cost
		High GDP content	
		Poor peritoneal membrane biocompatibility	
		Infusion pain	
		Local and systemic glucose exposure	
Icodextrin-containing; lactate buffered	Baxter	Hypersensitivity	Sustained ultrafiltration
		Low pH	Preservation of RRF
		Licensed for single daily use only	Hypertonic glucose replacement
		Lactate containing	Reduced hyperglycemia
			Improved short-term systemic hemodynamic profile
			Desirable effects on metabolic profile and body composition
Amino-acid containing; Nutrineal®	Baxter	Low pH	No GDPs
		Licensed for single daily use only (avoid exacerbation of uremic symptoms and acidosis)	Avoid systemic and peritoneal glucose exposure
			Peritoneal membrane protection
			Enhance nutrition

*GDP* glucose degradation product,* RRF* residual renal function

Icodextrin-containing solutions, an alternative to PDFs using glucose as the osmotic agent, are characterized by the generation of slow but sustained ultrafiltration [17, 18]. The ultrafiltration that takes place over a long dwell time is with a no or limited sodium-free filtrate, characteristic of “colloid” ultrafiltration. Conversely, when the dialysis solution has a high glucose concentration, the ultrafiltrate is generated by an osmotic crystalloid gradient that results in a more “pure water” filtrate, without sodium removal. Whereas the use of icodextrin-containing PDFs in older children produces results similar to those that occur in adults, a preliminary study has suggested that there is a risk of decreasing ultrafiltration over a long dwell time (i.e. 10–14 h) in infants and young children. It is therefore important to determine on an individual basis the optimal dwell time in infants/young children who are treated with icodextrin, in each case adapting it to the rapidity of the osmotic gradient loss over time [21, 23]. Whereas clinical experiences [17–19] in both adults and children have been favorable for the use of icodextrin during long dwell periods, giving rise to both increased ultrafiltration and improved clearances, the long-term experience in terms of peritoneal and systemic biocompatibility has yet to be determined, particularly in children [20, 21]. Indeed, icodextrin is associated with a risk for sterile peritonitis and cutaneous intolerance [17, 19].

Amino-acid-containing solutions also avoid glucose exposure and contain no GDPs [24]. Their application in dialysis patients with hypoalbuminemia has resulted in an improved nutritional state [19] in some studies. Little information exists on the use of these solutions in children [21]. Therefore, where available, their use remains restricted to several clinical conditions, such as during a peritonitis episode to counterbalance the enhanced protein loss, or possibly in the case of severe malnutrition.

All in all, we believe in the concept that enhanced PDF biocompatibility should improve patient outcome in terms of peritoneal membrane integrity. Therefore, the new biocompatible PDFs should be preferred over conventional PDFs for children receiving chronic PD. In addition, and despite their limited biocompatibility, the use of icodextrin-containing dialysis solutions in children is a particularly effective method to enhance ultrafiltration and even maintain patients on PD despite the presence of a high peritoneal membrane transport capacity.

## Determination of exchange fill volume

In adults on continuous ambulatory peritoneal dialysis (CAPD), the choice of an exchange fill volume is often limited to the prescription of a full dialysis bag of 2 or 2.5 L, without the capability of providing an individually adapted prescription based on body weight or BSA. In pediatric care, however, we must adapt the fill volume to the individual child considering the wide morphologic differences between infants and adolescents. A common question is: “What is the optimal fill volume for children on PD”? Twenty years ago, it was recommended that the fill volume be prescribed per kilogram of body weight, 30–50 ml/kg, lower in infants than in older children and lower at the initiation of dialysis so as to increase the likelihood of patient tolerance. Unfortunately, this approach led to prescriptions characterized by relatively small fill volumes. This limited fill volume concept also resulted in the false perception of the existence of differences in peritoneal permeability between children and adults, with a hyperpermeable peritoneal state presumably present in the youngest patients, especially in infants [25–27]. All of this could be explained by the “geometry of diffusion”, a concept that addresses the rapid equilibration of solute that occurs across the peritoneal membrane when using small fill volumes. This rapid equilibration has been repeatedly observed in infants prescribed a small fill volume over a relatively large peritoneal membrane surface area. Subsequent scaling of the fill volume by BSA (ml/m^2^), particularly in infants and small children, avoids this issue and also makes possible an accurate assessment of membrane transport capacity through use of the peritoneal equilibration test (PET) [9, 28–31]. Therefore, the fill volume prescribed for children should be scaled to BSA to preclude the provision of a fill volume that is too small and to prevent the development of functional hyperpermeability and apparent ultrafiltration failure due to a rapid loss of the glucose-related osmotic crystalloid gradient [9, 19].

In children older than 2 years, the presumed optimal fill volume should be increased stepwise close to the”upper limit” of 1,200–1,400ml/m^2^ for a nocturnal exchange in the prone position [9]. A small fill volume has been associated with an impaired statural growth rate [32] and with a discrepancy between urea and creatinine transport (urea – high; creatinine – low) [32–34]. Conversely, a fill volume that is too large may contribute to patient morbidity by causing complications such as pain, dyspnea, hydrothorax, hernia formation, gastroesophageal reflux with anorexia, and loss of ultrafiltration by enhanced lymphatic uptake [9, 29, 33]. Such morbidity, which is in part related to an elevated IPP, could also result in patient nonadherence. Therefore, increasing the fill volume above a peak volume could result in reduced efficiency rather than improvement [4, 35].

In infants younger than 2 years, the fill volume is based more on tolerance than on an optimal dialytic exchange volume; whereas an upper limit for fill volume of 800 ml/m^2^ (or 30–50 ml/kg body weight) [9] is often suggested, an individual clinical assessment of tolerance is of greatest importance. The bedside measurement of IPP can potentially be of assistance in this regard [11] (Table 3).

**Table 3:** Normal hydrostatic intraperitoneal pressure (IPP; cmH_2_O) related to the intraperitoneal fill volume [IPV; ml/m^2^ body surface area (BSA)] [4]

	IPP (cmH_2_O)	IPV (ml/m^2^ BSA)
In adults	13.4 ± 3.1	1585 ± 235
In children older than 2 years	5.2 ± 2.6	600 ± 50
	8.2 ± 3.8	990 ± 160
	14.1 ± 3.6	1400 ± 50

Procedure for measuring intraperitoneal pressure (IPP) [11]:

- The patient is invited to use the bathroom, if needed, to empty his or her bladder
- The patient is then placed at rest, lying completely flat
- The connection is made to the peritoneal system
- Any fluid present in the abdominal cavity is drained. A defined volume of PDF is then instilled
- The PD line is fixed vertically; there is no counterpressure in the distal part of the measurement tubing
- The zero level of the column (on the graduated scale) is set at the center of the abdominal cavity, on the medial axillary line
- The connection of the line to the patient is opened
- The level of the column of dialysis fluid in the PD line is read, with a scale graduated in centimeters after the height of the column stabilizes, initially after inspiration (IPP_insp_) and then after expiration (IPP_exp_)

Mean IPP (cmH_2_O) is determined as follows:

$${\text{Mean}}\;{\text{IPP}} = \frac{{IPPinsp + IPP\exp }}{2}$$

In summary, the prescribed fill volume should be low enough initially to be well tolerated by the patient but thereafter should be modified, potentially with the use of IPP [11] measurements, to achieve adequate ultrafiltration and urea purification, both of which are directly related to the size of the fill volume. The upper tolerated limit of IPP is 18 cmH_2_O, with the normal pressure being 7–14 cmH_2_O (Table 3).

## Determination of exchange dwell time

In CAPD the dwell times are long, and therefore, the major risk to dialysis efficiency is loss of the glucose-related osmotic gradient, resulting in ineffective ultrafiltration and dialysate reabsorption by the child. To limit this development, hypertonic dialysate is often prescribed despite the potential for enhanced peritoneal membrane toxicity related to the quantity of glucose and GDPs [19]. Whereas the use of dialysate with icodextrin as the osmotic agent is one means by which the time-related dialysate reabsorption can be limited in patients receiving CAPD, its application should be limited to one exchange per day [19, 20].

Most children, however, are treated with APD, a choice made to allow for better tolerance of a full peritoneal cavity, as occurs in the supine position, and to meet the need for short dwell times to limit dialysate reabsorption [9]. In most cases, nocturnal APD is also more compatible with a normal day life (i.e. uninterrupted schooling and parents’ work). It should be emphasized that short dwell-time exchanges that occur with APD are more appropriate for urea clearance than for phosphate clearance, important clinically because of the association between hyperphosphatemia and both osteodystrophy and cardiovascular disease [9, 12]. In fact, during a PET, the time needed to achieve a dialysate over plasma concentration ratio of 50–60% for phosphate is three to four times longer than it is for urea [9, 12]. Short dwell-time exchanges are, in turn, most likely to result in adequate ultrafiltration and urea purification (Kt/V_urea_) [36]. Long dwell-time exchanges, on the other hand, favor higher creatinine and phosphate clearance but with the risk of impaired ultrafiltration. Thus, whereas an exchange dwell time of approximately 1 h appears to be a typical choice for the initial APD prescription in children, it should subsequently be reevaluated and possibly modified based upon the needs of the individual patient, taking into consideration the patient’s growth, residual kidney function, peritoneal membrane function, and the desired clinical goals [5]: ultrafiltration (short dwell times) or phosphate purification (longer dwell times). The peritoneal membrane’s transport capacity can be determined clinically with tests such as the PET.

## Evaluation of peritoneal membrane function: solute transfer and net ultrafiltration

The most widely used test of peritoneal membrane function is the PET, developed and described by Twardowski in 1986 [37]. The test was designed to be performed during a 4-h dwell with a 2.27% glucose dialysis solution to evaluate small solute transfer across the peritoneal membrane and net ultrafiltration. In children, comparable studies have been performed with 2.5% glucose dialysis solution (North America, Japan) and 2.3% anhydrous glucose dialysis solution (Europe) [30, 31]. With this test, a patient’s membrane transport capacity is characterized as high, high-average, low-average, or low [9, 26, 30, 31, 38]. A test performed with a 3.86% glucose dialysis solution provides more accurate information on ultrafiltration capacity and facilitates assessment of sodium sieving, or the maximum dip in dialysate over plasma sodium concentration that typically occurs during the initial 30–90 min of dwell time [39]. This allows for evaluation of the free water transport capacity through the aquaporins, or water channels. In some children, the ultrafiltrate generated using a 3.86% glucose solution for the PET may, however, be a factor of discomfort or even intolerance. Although there is not a significant impact of using a 2.27% vs. 3.86% glucose dialysis solution on transport group characterization, the preceding exchange composition (e.g. icodextrin vs. glucose) and dwell time can influence results of the evaluation for protein transport during the PET [39, 40]. Therefore, it seems prudent to use the same PDF that is to be used for the PET the night before the PET is to be performed. In children, as discussed above, it is especially important to recognize that the size of the test exchange fill volume has a significant impact on the rapidity of solute equilibration and the PET results [9, 25, 26]. If the text exchange fill volume is scaled according to BSA, usually 1,000–1,100 ml/m^2^, the test results appear to be most valid and support the belief that there are no inherent differences in the peritoneal membrane transport capacity when comparing children of different ages or children to adults [26–28, 38]. Conversely, a fill volume that is too low can result in exchange hyperpermeability and rapid equilibration of solute across the peritoneal membrane because of the small fill volume that results in a high solute D/P (dialysate over plasma creatinine concentration) value and a high absorption rate of glucose, with a resultant decreased osmotic gradient [25, 26, 29]. In infants, however, the fill volume used for the PET is usually the clinically prescribed fill volume due to their limited tolerance of a high fill volume until approximately 2 years of age. When using a test exchange volume of 1,100 ml/m^2^ in children initiating PD, preliminary data suggest that a short PET, using only a 2-h dwell, may perform as well as the 4-h evaluation [41].

The ability to evaluate for ultrafiltration failure is also of major clinical importance [2, 39]. In the case of low drain volumes, a distinction must be made between catheter dysfunction, leakage of fluid either externally through the catheter tunnel or internally from the peritoneal cavity to the pleural space, and impairment of the peritoneal membrane. In fact, multiple membrane-related causes should be considered, which include the following:

1. Large peritoneal surface area in comparison with the size of the fill volume, the result of either too low a prescribed fill volume or too large a vascular surface area secondary to hyperperfusion (e.g. GDP-induced neoangiogenesis)
2. Impaired free-water transport as a result of aquaporin dysfunction
3. High lymphatic absorption associated with a marked elevation of IPP
4. Limited peritoneal surface area available for exchange, as might occur with postinfectious or postsurgical adhesions, peritoneal fibrosis, or peritoneal sclerosis

As mentioned above, free-water transport characteristically occurs during the first hour of a PET conducted with a 3.86% glucose dialysis solution and contributes about 40–50% of this early ultrafiltration [39]. When impaired ultrafiltration capacity exists, one would expect to see little or no dip of the D/P sodium as a result of impaired free-water clearance.

The personal dialysis capacity (PDC) test [42], which is based on the three-pore model [16] and which is validated in adults and in children [43], describes the peritoneal membrane transport characteristics by functional parameters, derived from data obtained following several exchanges of different duration and different glucose concentrations performed over an entire day. These parameters are: (a) the surface area over the diffusion distance, which represents the total vascular surface area available for diffusion and is thought to be comparable to the D/P value of the PET; (b) the reabsorption parameter, primarily representing lymphatic flow; and (c) the large pore flow, which is reflected by the loss of protein/albumin from the blood to the peritoneal cavity. A large pore flow that is higher than expected according to the total vascular surface area assessment appears to be the most sensitive sign of an inflamed peritoneal membrane [44] and is correlated with an increased mortality risk. Use of the PDC or placement of dextran in the dialysis solution and subsequent analysis is the best means by which to evaluate for enhanced lymphatic absorption. Despite all the information given by the PDC test, its use appears to be limited to a few pediatric dialysis centers, predominantly those involved in PD-related research.

Dialysate cancer antigen 125 (CA125) concentration within the dialysis effluent can be used as a marker of mesothelial cell mass and thereby as a membrane integrity parameter, but no clear correlation between these results and peritoneal transport parameters have been established [8, 19].

Finally, the dialytic removal of solute can be quantitated by clearance (K) or mass transfer area coefficient (MTAC) calculations. For urea, the clearance is scaled to the urea volume of distribution (V), which is equivalent to total body water. At present, V may be estimated using gender- and height-adapted formulas for children [45]. The weekly Kt/V_urea_ is derived from the daily value and in practice is the principal quantitative factor used to characterize PD adequacy [46]. Creatinine clearance (K_creat_) is another clearance measure and is calculated in milliliters per minute and then converted to weekly K_creat_ and expressed in liters per 1.73 m^2^ BSA. The residual kidney function is calculated as the mean of creatinine and urea renal clearances and should be integrated in urea and creatinine dialytic adequacy parameters.

The MTAC characterizes the dialytic removal of solute and is a measure that is essentially independent of dialysis mechanics (e.g. exchange volume or dialysate glucose concentration). Different formulas to calculate the MTAC are available [30, 47–49].

## Peritoneal dialysis efficiency: adequate prescription before optimal adequacy

Ultrafiltration, water and sodium balance, and blood purification for more than just urea are key treatment goals of the PD prescription process. Overhydration is an important problem in children receiving PD and is most notable when the residual kidney function-associated diuresis is substantially decreased. A number of factors may contribute to sodium and water retention. Dietary compliance in terms of sodium and water consumption and mechanical problems such as catheter dysfunction should be excluded before implicating PD inadequacy as being the result of either membrane failure or an inappropriate or ineffective prescription. As mentioned previously, a PET conducted with a 3.86% glucose dialysis solution can determine the permeability of the peritoneal membrane to solute, the amount of drained ultrafiltration (net ultrafiltration) over the 4-h dwell period, and the maximum dip in D/P sodium, which reflects sodium-free water as a reflection of aquaporin function. If the patient is found to be a rapid transporter, the result of either a large peritoneal surface area or a prescribed fill volume that is too low, he or she may benefit from altering the dialysis prescription by increasing the fill volume as tolerated and/or by shortening of the dwell time during APD to improve ultrafiltration. Children with a decreased sodium-free water transport and no dip in D/P sodium after a 1- to 2-h dwell will not benefit from the use of a higher dialysate glucose concentration. On the other hand, they may enhance their ultrafiltration capacity by using a long exchange with icodextrin, as might occur during the day with APD or at night for those receiving CAPD. If enhanced lymphatic reabsorption is a strong consideration, an indirect therapeutic adjustment is to decrease the fill volume, with the hope that a decreased IPP may in fact reverse the propensity for reabsorption.

Urea removal scaled for the urea volume of distribution, Kt/V_urea_, is the recommended parameter to use as a surrogate for adequate dialysis, at least in the case of CAPD [46]. Although little comparable information exists for APD, Kt/V_urea_, and sometimes K_creat_, is regularly measured for patients on this modality as well [46, 50]. Historically, both Kt/V_urea_ and K_creat_ were the recommended adequacy parameters for patients receiving CAPD, and it was proposed that the ratio of the two parameters should be 1 to 30 (e.g. Kt/V_urea_ 2.4 L/week and K_creat_ 72 L/week) [9]. Somewhat surprisingly, the same correction factor has been used in general pediatric care when adjustments are made in drug prescription from milligrams per kilogram body weight to milligram per meter squared BSA. This is in fact the bridge from a volume of distribution such as the vascular blood compartment, to a whole cellular distribution, such as the cell membrane area. Maintaining this ratio of urea to creatinine purification also appears to be significant from a physiologic perspective, as it pertains to both cellular purification (e.g. uremic toxin compartment) and total body-water purification (e.g. urea volume of distribution).

When a discrepancy in the clearance parameters exists and the Kt/V_urea_ is deemed adequate and the K_creat_ inadequate [32, 51], this could be the result of a hyperpermeable peritoneal membrane state or, as mentioned previously, a fill volume that is too low, both of which result in significantly greater removal of urea vs. creatinine across the peritoneal membrane. As also noted above, impaired growth has been reported to be [30] associated with a clearance determination characterized by an adequate Kt/V_urea_ and a low K_creat_. Thus, whereas it is often difficult to reach both parameters of adequacy and the most recent National Kidney Foundation Kidney Disease Outcomes Quality Initiative (KDOQI) guidelines on PD adequacy recommend the preferential use of Kt/V_urea_ as the adequacy parameter, every effort should be made in the prescription process to achieve the urea over creatinine purification ratio [9, 34, 51]. This may be most pertinent in the APD population, for whom K_creat_ targets have recently been published and in whom the relationship between K_creat_ and Kt/V_urea_ is much more variable than it is with CAPD [50].

Whereas the removal of urea by PD is proportional to the daily amount of PDF prescribed, the efficiency of creatinine and, possibly most importantly, phosphate removal are related to contact time and clearance. In practice, an enhanced fill volume will impact urea removal, whereas an extended dwell time is necessary for optimal phosphate removal [12, 36, 52]. Therefore, efficiency of the long daytime exchange in patients receiving APD is exceptionally important, and in some cases, the use of icodextrin during the day, which characteristically counters the absorption of dialysate over the extended hours, may be most ideal and complements the short dwells that characterize cycler dialysis overnight. A new generation of cyclers that currently offers the possibility of changing fill volume, dwell time, and dialysate composition for each cycle of the overnight cycling procedure has great potential for further contributing to improved dialysis efficiency.

Residual kidney function may have a significant impact on patient outcome and can assume greatest significance in situations in which target solute clearances fail to be achieved solely by the dialysis process [53, 54]. Efforts to preserve residual kidney function include the prevention of nephrotoxic insults such as exposure to radiocontrast dye, aminoglycoside antibiotics, and extracellular fluid (ECF) volume depletion [46]. Preferential use of an angiotensin-converting enzyme inhibitor/angiotensin receptor blocker for blood pressure control has also been proposed [55].

Finally, it has been recommended that in children, the prescription should be individualized and that the overall clinical status of the patient is an outcome parameter that is as important to monitor as Kt/V_urea_ and K_creat_ when determining the ideal patient prescription. True prescription modification may be necessary to achieve target clearances if there have been repeated bouts of peritonitis and a resultant decreased dialysis clearance, or a loss of residual kidney function. Likewise, growth and nutritional status can be used as parameters to evaluate efficacy of the dialysis purification process. The recent report by Fischbach et al. on these clinical measures has, in turn, prompted questions about whether the “best” dialysis modality is daily PD or daily and intensified hemodiafiltration [56]. In most instances, the lowest dialysis glucose concentration should be used with PD, despite the fact that the absorption of glucose may theoretically provide some caloric benefit to the anorectic patient; it should not replace adequate nutrition by being included in the dietary prescription. It is also possible that the use of PDFs with a high glucose concentration may result in hyperglycemia and potentially limit the patient’s appetite.

There is no uniformed optimal PD prescription for all children, nor is there an optimal PDF. However, if the new, more physiological, dialysis solutions are clearly shown to improve peritoneal membrane function in humans [19, 21, 57], their preferential use in children should complement improvements in technology. They will also enhance our greater understanding of factors that alter the structure and function of the peritoneal membrane to improve the prescription process for the benefit of the pediatric patient who has a lifetime of ESRD care before them.

## Questions

(Answers appear following reference list)

1. Choice of PDF should take which of the following into consideration:
  A. Inflow pain is primarily related to PDF osmolality
  B. GDPs induce peritoneal vascular neoangiogenesis with diabetiform changes
  C. Dialysate glucose concentration is associated with little or no membrane toxicity
  D. Short dwells, with reduced PDF to peritoneal membrane time as prescribed in APD, limit PDF related-membrane toxicity
2. Peritoneal fill volume is characterized by which of the following:
  A. Prescribed fill volume should be determined solely by the child’s perceived clinical tolerance
  B. Pain during peritoneal dialysis is only related to the size of the fill volume
  C. Measurement of IPP is an objective parameter of the tolerance of a fill volume
  D. Fill volume that is too small will induce hypopermeability
3. Which of the following accurately describes the process of peritoneal dialysis:
  A. Dialysis solute removal is impacted by the size of the fill volume
  B. Dialysis solute removal is impacted by the prescribed dwell time
  C. Short dwell time improves ultrafiltration capacity
  D. Long dwell time improves phosphate removal
  E. Long dwell time is a risk factor for PDF reabsorption
  F. All of the above
4. Reduced drained volume could be related to all but which of the following:
  A. Large peritoneal surface area
  B. Small peritoneal surface area
  C. Technical problems with the PD catheter
  D. Low IPP
  E. Hyperpermeable peritoneal membrane
5. Which of the following characterizes the peritoneal equilibration test (PET):
  A. A 1.36% glucose PDS should be used to assess peritoneal membrane permeability and ultrafiltration capacities
  B. PET fill volume should be 1,100 ml/m^2^ BSA only in infants
  C. It helps characterize peritoneal membrane permeability
  D. It should be performed successively with all prescribed PDS, even with icodextrin
6. An important principle of dialysis efficiency is:
  A. The higher the Kt/V_urea_, the better the child’s outcome
  B. There is no peak fill volume for dialytic removal of urea
  C. Dialytic removal of phosphate is fill-volume and dwell-time dependent
  D. Free-water ultrafiltration is unrelated to the function of the ultrasmall pores
